# Reports of Montreal Veterinary College

**Published:** 1883-04

**Authors:** 


### MONTREAL VETERINARY COLLEGE.

The examinations of this institution, which have been in progress during the last ten days, were concluded March 29th by the final oral examination by the Board of Examiners appointed by the Council of Agriculture consisting of the following gentlemen: F. S. Billings, M. V.; Williamson Brydon, V. S., Boston, Mass.; C. J. Alloway, V. S., Montreal; J. A. Couture, V. S., Quebec; A. McCormack, V. S., Ormstown; Chas. Levesque, Berthier en haut, and Dr. Geo. Leclerc. The following gentlemen were present and assisted in the exercises: Hon. G. Ouimet, Commissioner of Public Instruction, in the chair, supported by Prof. R. P. Howard, Dean of the Medical Faculty of McGill University; Prof. Beaudry, representing Victoria University; J. M. Browning, Vice-President of the Council of Agriculture; Geo. Leclerc, Secretary, and Rev. Father Pilot, Mr. W. S. Blackwood, A. Sommerville and Casgrain, the Educational Committee of the Council; Prof. Osler, Prof. Daubigney, Dr. Sutherland Baker, and a large number of visitors.

Hon. Mr. Ouimet spoke of the good work done by the college, and proceeded to distribute prizes and diplomas, complimenting the recipients on their success.

The following students enregistered during the past session:

Wm. B. Abbey, New Bedford, Mass.; M. G. Blanchard, N. S.; A. A. Keys, Ont.; W. G. Johnston, P. Q.; Geo. Sangster, Q.; W. P. Robins, Q.; A. W. Clement, Mass.; W. F. Scott, Q.; James Brodie, Q.; C. J. Davis, P. Q.; C. D. Bancroft, Q.; E. P. Ball, Q.; B. A. Pomeroy, Q.; C. L. Morin, Q.; M. Piche, Q.; J. A. Bishop, Q.; R. W. Hopper, Q.; H. K. Durfee, Mass.; Charles G. Lamb, Mass.; Wm. Bell, Ont.; H. C. Kingman, Mass.; E. C. Crevier, Q.; A. Beauchamp, Q.; W. H. Klock, Ont.; John Magor, Q.; John Henry, Iowa; A. E. Cross, Q.; J. T. O'Donnell, Mass.; C. Drouin, Q.. J. Labelle, Q.; T. G. Brosseau; W. S. Renner; O. D. Fortin, Q.; J.

Lanctot, Q.; R. Lapointe, Q.; Fred. Paquin, Q.; H. Pilon; C. P. Drake, Q.; A. C. Rouif, Q.; P. A. Gindun; T. Beauchamp; W. P. Mass.; Ed. W. Hoare, Man.; W. S. Mahon, W. I.; J. W. Sparks, Mass.

The following students passed in the undermentioned subjects in order of merit:

Botany, Prof. J. W. Dawson, McGill College—Scott, Durfee, Lamb, Mahon, Sparks, Magor, Keys and Abbey.

Chemistry, Prof. Girdwood, McGill College—Blanchard, Kingman, Ball, Davis, Cross and Mayo.

Physiology, Prof. Osler, McGill College—Blanchard, Ball, Kingman.

Materia Medica, Dr. James Bell, Veterinary College—Kingman, Bancroft, Blanchard, Ball, Cross, Davis, Klock.

Anatomy, M. C. Baker, V.S., Professor—Brodie, Bell, Clement, Pomeroy, O'Connell, Henry, Duncan, Robins, Bancroft.

Practice of Veterinary Medicine and Surgery and General Pathology, D. McEachran, F.R.C.V.S., Professor—Bell, Clement, Brodie, Henry, Pomeroy, Duncan, O'Connell, Bancroft and Robins.

Physics, Prof. Girdwood, McGill College—Lamb, Durfee, Scott, Hoare, Mahon, Magor, Abbey, Keys and Sparks.

FRENCH CLASSES.

Botany, Prof. Roy, Victoria College—A. Beauchamp, T. Brosseau, Fortin, Lapointe, Piche, Rouif, Turcot.

Physiology, Prof. Beaudry, Victoria College—Morin, Labelle.

Chemistry, Prof. Munier, Victoria College—Morin, Labelle.

Obstetrics, Prof. M. Daubigny, V.S., Veterinary College—Crevier, Paquin, Drouin, Pilon.

Materia Medica, M. Daubigny, Veterinary College—Paquin, Crevier, Drouin, Pilon.

Anatomy, M. Daubigny, Veterinary College—Paquin, Crevier, Drouin, Pilon.

Practice of Veterinary Medicine and Surgery, and General Pathology, M. Daubigny, V.S.—Paquin, Crevier, Drouin, Pilon.

The following candidates passed the examinations successfully and received the diploma of the College: Messrs. Brodie,

Bell, Clement, Crevier, Henry, O'Connell, Pomeroy, Paquin and Robins.

PRIZES.

The following prizes were awarded English classes :

Seniors—best general examination in all subjects—Silver medal, the gift of the Council of Agriculture, won by Jas. Brodie.

Practice of Medicine and Surgery—Valuable Microscope, the gift of David Morrice, Esq., won by Wm. Bell ; second prize, A. W. Clement.

Anatomy—First prize, James Brodie ; second prize, Wm. Bell.

Practical Dentistry—Instruments, the gift of Williamson Dryden, Esq., V.S., won by H. J. O'Connell.

Juniors—Materia Medica—H. C. Kingman.

Anatomy—First prize, H. C. Kingman ; second prize, M. G. Blanchard.

Practice of Medicine and Surgery—First prize, H. C. Kingman ; second prize, E. P. Ball.

Botany—Gift of Prof. Dawson, won by W. F. Scott.

FRENCH CLASSES.

Best general examinations, silver medal, the gift of the Council of Agriculture, won by Fred. Paquin ; second prize, valuable instruments, the gift of L. H. Massue, Esq., M. P., President of Council of Agriculture, won by E. C. Crevier.

Obstetrics, the gift of M. Daubigny, won by E. C. Crevier.

Anatomy, the gift of Geo. Leclerc, M. D., won by Fred. Paquin.

At the conclusion of the exercises Mr. Billings, of Boston, complimented the Dominion on having in the Veterinary College of Montreal a gentleman at its head who, more than any one else, combined in his teaching and daily life science and practice, and whose honest efforts were devoted to the elevation of the profession.

Prof McEachran, was next called upon, and took advantage of the occasion to compliment the students on their honest hard work during the session, congratulated the successful and sympathized with those who had failed in passing the severe examinations to which they had been subjected. He pointed out advantages of a high standard of education on account of the rapid progress of the profession. No profession holds out, said he, more brilliant prospects than did the one they now entered, but they must not expect to gain success without a struggle, but by perseverance they were sure to succeed. He wished them all success. He next paid a high tribute to McGill and Victoria Colleges, thanked those gentlemen who gave prizes, especially David Morrice, L. H. Massue, Williamson Bryden and others, also the examiners who had come long distances to assist them, the Council of Agriculture and the Government of Quebec for their valuable support, and those gentlemen who had assisted them at the examinations.

Prof. R. P. Howard, Dean of the Medical Faculty of McGill, next addressed the students and graduates, complimenting them and their teachers on their success, and indicated that the Faculty seeing the necessity for extending the field of study had decided to add to their College a chair of comparative pathology, which they hoped soon to see accomplished. He could say for the Medical Faculty that they had always great pleasure in doing all they could to assist the Veterinary College, which was looked upon by them as one of the most useful institutions in the province.

Prof. Beaudry next followed in a similar strain for Victoria College.

Dr. Osler, J. M. Browning and others followed in eulogistic remarks of the College and its Principal.

The proceedings terminated by a vote of thanks to Hon. Mr. Onimet.

A meeting of the Veternary Medical Association was held immediately after, when the diploma was conferred on Wm. Bell, Jas. Brodie, A. W. Clement, E. C. Crevier, Jno. Henry, T. J. O'Connell, B. A. Pomeroy, Fred. Paquin, Paul Paquin, also on Mr. F. S. Billings and Prof. Daubigny.

---

Account of the examination of this College was received too late to be inserted under the proper heading.

